# The association between sensor-based assessments of daily physical activity patterns and physical fitness in older adults: a systematic review and meta-analysis

**DOI:** 10.1186/s11556-025-00381-y

**Published:** 2025-09-30

**Authors:** Shan Su, Jing-Yuan Liu, Clare Chung-Wah Yu, Shirley Pui-Ching Ngai, Siu-Ngor Fu

**Affiliations:** 1 Department of Rehabilitation Sciences, The Hong Kong Polytechnic University, Kowloon, Hong Kong S.A.R.; 2 Department of Applied Social Sciences, The Hong Kong Polytechnic University, Kowloon, Hong Kong S.A.R.

**Keywords:** Older adults, Physical activity, Physical fitness

## Abstract

**Background:**

This study aimed to investigate the association between sensor-based assessment of daily physical activity patterns and physical fitness among older adults by meta-analyses of relevant studies.

**Methods:**

A systematic search was conducted across six databases (PubMed, CINAHL by EBSCOhost, Web of Science, PsycInfo by ProQuest, Embase, and Scopus) from inception until January 18, 2025. Manual searches of reference lists and Google Scholar were also performed, utilizing predefined keywords to identify observational studies with bivariate association analyses. Joanna Briggs Institute Critical Appraisal Checklist for Cross-Sectional Studies was employed to assess study quality. The association analyses were further categorized based on the characteristics of daily activity (sedentary, light, moderate-to-vigorous) and physical fitness (e.g., grip strength, knee muscle strength, leg power, walking endurance, mobility function, and gait speed).

**Results:**

Thirteen cross-sectional studies were included in the meta-analyses. On average, older adults spent 78% of their day sedentary, 15% in light-intensity activity, and 7% in moderate-to-vigorous activity. Sedentary behavior was linked to lower physical fitness ($r=-0.10$ to $-0.47$), while moderate-to-vigorous activity was linked to better fitness ($r=0.21$ to 0.43); light-intensity activity showed only weak and selective associations with better mobility function and gait speed ($r=0.20$ to 0.26).

**Conclusion:**

This review shows that while older adults spend much of their day sedentary, both moderate-to-vigorous and light-intensity physical activity are linked to better physical fitness. Promoting light-intensity activities may be a practical strategy to support mobility and independence, especially for those unable to meet higher activity guidelines. However, recommendations should remain cautious, as the evidence supporting an association between light-intensity physical activity and physical fitness is currently weak.

**PROSPERO registration:**

The protocol of this systematic review was registered on PROSPERO (Registration number: CRD42023471302).

**Supplementary Information:**

The online version contains supplementary material available at 10.1186/s11556-025-00381-y.

## Introduction

Amidst the global demographic shift toward aging populations, older adults face increasing risks of age-related health conditions, including chronic diseases, functional decline, and multimorbidity [1]. To mitigate these challenges, the World Health Organization (WHO) has established physical activity guidelines aimed at reducing the risk of disability and comorbidities in older adults. Specifically, the WHO advises at least 150 to 300 minutes of moderate aerobic exercise or an equivalent combination of moderate- and vigorous-intensity activity weekly, which is associated with various health benefits [2–6]. Recent updates to WHO guidelines emphasize that every movement counts and advocate minimizing sedentary time, even for individuals unable to meet formal exercise targets [2]. These benefits are thought to be largely related to improvements in physical fitness—a multidimensional construct encompassing cardiorespiratory endurance, musculoskeletal strength/power, balance, and functional mobility [7, 8]. These domains collectively determine an individual’s capacity to perform daily activities independently, with moderate-to-vigorous-intensity exercise being the conventional approach linked to enhanced fitness [9, 10]. However, current evidence suggests alarmingly low compliance rates, with fewer than 15% of older adults meeting recommendations [11], while over half exhibit sedentary behaviors exceeding 8.5 hours daily [12]–a pattern that may both contribute to and result from age-related functional decline, highlighting the complex bidirectional relationship between sedentary behavior and functional decline.

In contrast, older adults engage in substantial light-intensity physical activity, averaging 73 minutes daily—approximately twice that of younger populations [13]. Emerging evidence highlights an association between light-intensity physical activity and better cardiometabolic health, although its association with fitness appears limited [14–16]. Among older adults, recent studies associate engagement in light-intensity physical activity with enhanced gait speed, balance and mobility ability [17, 18], which could reflect the possible correlations between consistent low-level activity and better neuromuscular coordination and functional capacity in the context of age-related decline. While these findings suggest potential associations between light-intensity physical activity and physical fitness, considerable methodological heterogeneity in participant characteristics, physical activity measurement methods, and outcome assessments across studies limits the ability to establish firm conclusions or generalize findings.

Advances in sensor-based physical activity assessment provide objective, detailed data on activity patterns and intensities, overcoming limitations of self-reported measures [19, 20]. Despite this, the relevance and application of sensor-based methodologies in studying the associations between physical activity and fitness among older adults have not been comprehensively synthesized. This gap is particularly important given that many existing longitudinal studies rely on self-reported data or focus on broader activity categories [21, 22]. While longitudinal studies have documented the influence of physical activity on health outcomes over time, cross-sectional studies remain crucial for identifying current associations between sensor-based assessments of daily physical activity and physical fitness. Cross-sectional evidence can provide valuable snapshots of activity patterns and their correlations with fitness outcomes, informing hypothesis generation and guiding future longitudinal research and interventions.

Therefore, this systematic review aimed to synthesize cross-sectional studies that examine the associations between sensor-based assessments of physical activity and physical fitness among older adults. By focusing on sensor-based measurement methods and current activity-fitness relationships, this review seeks to clarify existing evidence, address methodological heterogeneity, and highlight areas for future research to support healthy aging and functional independence.

## Methods

### Study design

This systematic review was conducted and reported in accordance with the Preferred Reporting Items for Systematic Reviews and Meta-analyses (PRISMA) Checklist (Supplementary A) [23].The protocol was registered on PROSPERO (Registration no. CRD42023471302).

### Search strategies

A comprehensive search of six electronic databases (PubMed, CINAHL by EBSCOhost, Web of Science, PsycInfo by ProQuest, Embase, Scopus) was conducted to identify relevant studies published before January 18, 2025. The search strategy included the key words related to “physical activity”, “physical fitness” and “sensor-based assessments” (Table 1, Supplementary B). Additionally, manual searches of reference lists of included studies and Google Scholar were performed to identify any further relevant publications.

**Table 1:** Key words for searching

Domain	Key terms
Physical activity	Physical activit*; physical activity level*; level of physical activity; Daily physical activit*; leisure physical activit*
Physical fitness	Aerobic capacity; cardiopulmonary fitness; walk test; balance; strength; endurance; physical performance; physical capacity; physical function
sensor-based assessments	Watch; acceleromet*; actigraph; tactical; sensewear; inclinom*; activpal; activity monitor; pedometer

### Study selection

**Fig. 1:**
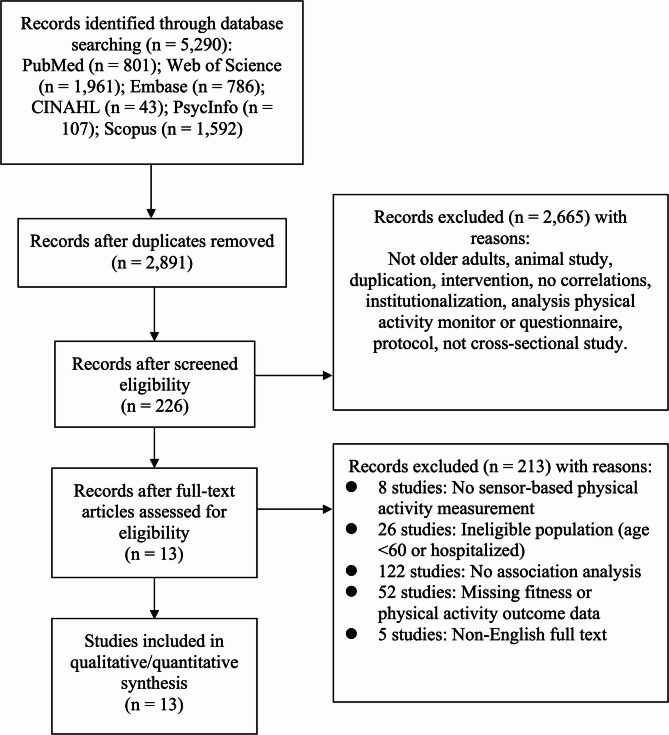
PRISMA flow diagram

Two independent researchers (SS and LJY) screened the titles and abstracts to identify studies meeting the predetermined inclusion and exclusion criteria (Supplementary C). The inclusion criteria were: (1) cross-sectional studies published in English, (2) older adults aged 60 years or above, and (3) studies reporting associations between any component(s) of daily physical activity (sedentary behavior, light-intensity physical activity, and/or moderate-to-vigorous-intensity physical activity) and physical fitness. Studies analyzing partial components (e.g., sedentary time alone) were included to maximize evidence synthesis, with analyses stratified by reported intensity domains. Studies were excluded if they were not full-text articles, if the age range of the participants was unclear, if they involved hospitalized individuals or participants requiring physical assistance from another person to walk, or if they were review articles or commentaries. The details of the included studies are provided in Table 2 and the reasons for exclusion are stated in Fig. 1.

### Definition and threshold of physical activity

The intensity of physical activity refers to the amount of energy required to perform an activity over time and is measured in metabolic equivalent task (MET). One MET is defined as an oxygen consumption rate of 3.5 ml of oxygen per kilogram of body mass per minute ($\mathrm ml.kg^{-1}.min^{-1}$) [24]. Based on this metric, sedentary behavior is classified as 1.5 METs or less, light-intensity physical activity ranges from 1.6 to 3 METs, and moderate-to-vigorous-intensity physical activity is categorized as 3 METs or more. Physical activity was typically classified using the threshold defined by counts per minute. Specifically, 0-99 counts per minute indicate sedentary behavior, 100-2019 counts per minute correspond to light-intensity physical activity, and $\ge 2020$ counts per minute represent moderate-to-vigorous-intensity physical activity [25]. Participants are required to wear the physical activity monitor during waking hours or throughout the day, excluding water-based activities. Valid data are obtained from participants who wear the monitor for at least three days, with a minimum of 8 hours per day to ensure accurate average readings.

### Data extraction and quality assessment

Data extraction was performed by two independent researchers (SS and LJY). Any discrepancies were resolved through consensus; if consensus could not be reached, a third author (CY) was consulted for adjudication. The following information was extracted from the selected studies: journal details (author names, publication year), sample information, participants’ source, relevant outcomes, and correlation coefficients.

The quality of the retrieved studies was assessed using the Joanna Briggs Institute (JBI) quality appraisal tool for cross-sectional studies by two reviewer authors (SS and JYL) independently [26]. This checklist comprises eight criteria: (1) clear eligible criteria; (2) detail description of study subjects and settings; (3) valid and reliable methods for measuring exposure; (4) objective and standardized criteria for measuring conditions; (5) identification of confounding factors; (6) strategies for managing confounding factors; (7) valid and reliable method for measuring outcomes; and (8) appropriate statistical analysis. Each criterion was scored as 1 for “yes” and 0 for “no” or “unclear”. The quality score for each study was calculated by dividing the actual total score by the expected total score, with the quality of studies categorized as low ($<50\%$), medium (50%−69%), or high ($\ge 70$%). Any disagreements during the quality appraisal were resolved through evidence-based discussions with another review author (CY). Individual study scores are summarized in Table 3.

### Statistical analysis

Statistical analyses were conducted using RStudio and related R packages, including “meta”, “metafor”, “forestplot” [27–30]. To synthesize effect sizes for correlation coefficients, data were presented as Fisher’s Z scores along with their corresponding 95% confidence interval (CI), which were derived from correlation coefficients [31]. The pooled Fisher’s Z value was subsequently converted back to a correlation coefficient. The predetermined threshold for statistical significance was set at $p < 0.05$. For outcomes not included in the meta-analysis, a narrative synthesis was employed to provide a qualitative summary of findings. A negative association indicates that increased time spent in the activity correlates with poorer physical fitness, while a positive association suggests that increased time is linked to improved physical fitness.

Studies were grouped based on the average activity reported in the study samples (active: $\ge 150$ minutes/week; inactive: $< 150$ minutes/week or described as sedentary). This classification was used to investigate whether the relationship between physical activity (particularly light-intensity or sedentary time) and physical fitness varies by participants’ baseline activity level.

Heterogeneity among the included studies was assessed using I-square ($\mathrm I^{2}$) statistic, which measured the proportion of variation across studies that exceeds what would be expected by chance alone. $\mathrm I^{2}$ values above 50% indicate high heterogeneity among studies. A random-effects model was utilized as a conservative approach to estimate the statistical significance of the effect [32]. Publication bias and small study effects were evaluated by funnel plot visually and by conducting Egger’s test.

## Results

### Screening results

The search from electronic databases retrieved 5,290 articles as shown in Fig. 1. After the duplicates were removed, 2891 articles remained. A further 2665 articles were excluded after screening for titles and abstracts. Consequently, a total of 226 articles were accessed for eligibility. Ultimately, 13 studies reporting associations between physical activity domains and physical fitness were included in the meta-analyses [33–45].

### Study characteristics

**Fig. 2:**
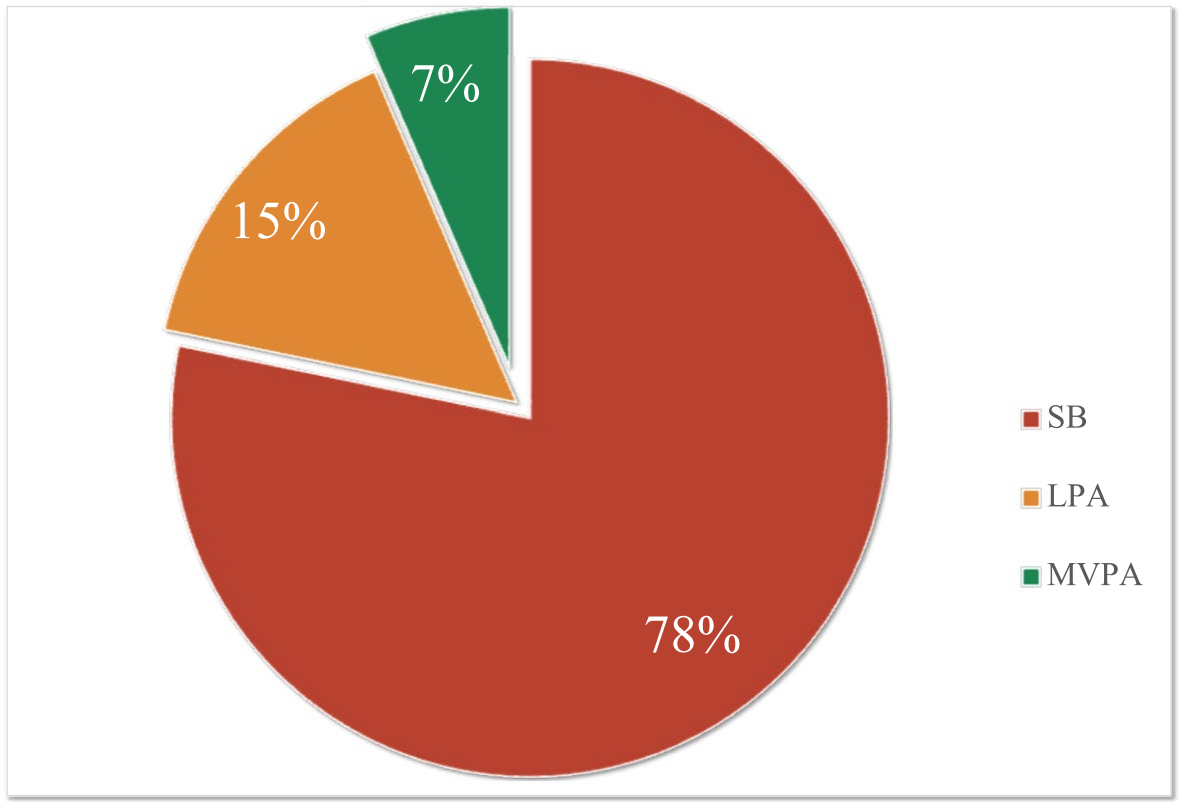
Pie chart of daily physical activity distribution. (Abbreviations: LPA = light-intensity physical activity; MVPA = moderate-to-vigorous-intensity physical activity; SB = sedentary behavior)

Table 2 presents the summarized characteristics and findings of the included studies. The meta-analysis yielded a mean participants age of 75.0 years across a cumulative sample size of 4921 individuals. Device wear-time data, reported in several studies, indicated an average daily usage of 12-15 hours [33, 34, 39, 40, 42]. Sedentary behavior averaged between 458 and 801 minutes per day [33, 34, 37, 39, 40, 42, 44], light-intensity physical activity spanned 57 to 291 minutes per day [33, 34, 36, 37, 39, 40, 42, 44, 45], and moderate-to-vigorous-intensity physical activity ranged from 10 to 87 minutes per day [33–45]. Figure 2 aggregated these activity levels daily, revealing that participants spent an average of 692 minutes per day in sedentary behavior (78% of total wearing time), 135 minutes in light-intensity physical activity (15% of total wearing time), and 57 minutes in moderate-to-vigorous-intensity physical activity (7% of total wearing time). Physical fitness was measured with different scales. The chair stand test, which measures leg power, was utilized in 7 studies [34, 35, 37, 38, 40–42]. Seven studies adopted the timed up-and-go test (TUG) to measure participants’ function of mobility [34, 36–41]. And the six-minute walk test was utilized in 5 studies for walking endurance [33, 34, 37, 38, 41], and 4 to 10-meter walk test were performed in 6 studies to measure the gait speed [33, 35, 41–44]. Knee extension/flexion strength in three studies [43–45] and grip strength in two studies [39, 43] were measured using a dynamometer.

**Table 2:** Summary of included studies

Study	Country	Study population (Age; no. (%female); BMI)	Physical activity	Physical fitness
Abe, 2012 [45]	Japan	Older women (52-76 y.o.), without regular resistance exercise program for at least 5 years 65.7±6.4 48 (100%) 22.4 ± 2.6	Lifecorder-EX accelerometer Left or right hip Consecutive 30 days LPA (min/d): 59.4±20.8 MVPA (min/d): 24.10±11.93	Isometric KE (Nm): 105±25 Isometric KF (Nm): 45±9
Aoyagi, 2009 [43]	Japan	Older adults (65-84 y.o.), with functional independence, absence of chronic conditions (particularly severe disease and/or pain) 72.6±4.6 170 (55.3%) 23.3±3.3	Pedometer (Uniaxial) Left side of the body 1 year MVPA (min/d): 17.3±11.9	Isometric KE (Nm/kg): 1.34±0.37 Grip strength (Newton): 268±83 5-meter usual walk (m/s): 1.40±0.22 5-meter fast walk (m/s): 2.0±0.4
Davis, 2014 [42]	UK	Independently ambulatory older adults ($\ge 70$ y.o.) in urban communities 78.1±5.8 217 (50%) 27.3±4.9	ActiGraph (Triaxial) Waist Consecutive 7 days Wear time (min): 864.0±84.0 SB (min/d): 616.3±87.8 LPA (min/d): 234.8±46.8 MVPA (min/d): 13.0±18.7	SPPB-chair rise: 2.7±1.3 SPPB-balance: 3.6±0.8 SPPB-gait: 3.5±0.8
Duck, 2019 [36]	USA	Older adults ($\ge 65$ y.o.), with independently ambulatory, in a rural community $\ge 65$ 101 (78%) NR	GT3X ActiLife accelerometers Right hip Consecutive 7 days LPA (min/d): 114.2±55.9 MVPA (min/d): 11.4±13.1	TUG (s): 9.11±2.93
Gerdhem, 2008 [44]	Sweden	Older women ($\ge 80$ y.o.), from an Osteoporosis Prospective Risk Assessment study 80.1±0.1 57 (100%) 25.5±3.9	Accelerometer_MTI (Uniaxial) Right hip Consecutive 7 days SB (min/d), median (IQR): 630 $(569-682)$ LPA (min/d), median (IQR): 68 $(44-100)$ MVPA (min/d), median (IQR): 13 $(6-23)$	Isometric KF (Nm): 117±37 Isometric KE (Nm): 246±71 30-meter fast walk (m/s): 1.2±0.3
Ofei-Dodoo, 2018 [38]	USA	Older women ($\ge 65$ y.o.), from Senior Centers in communities 75.0±7.2 101 (100%) NR	Kenz Lifecorder accelerometer Waist Consecutive 14 days MVPA (min/d): 13.9	6MWT (m): 534.98±110.54 5STS (s): 12.68±3.44 TUG (s): 6.32±1.86
Orwoll, 2019 [35]	USA	Community-dwelling older men ($\ge 65$ y.o.), able to walk unaided, without major diseases 78.9±5.1 2741 (0%) 27.1±3.7	SenseWear Pro Armband Right triceps Consecutive 7 days MVPA (min/d): 86.9±57.5	5STS (s): 11.52±3.54 6-meter usual walk (m/s): 1.14±0.23
Osuka, 2015 [40]	Japan	Community-dwelling older adults ($\ge 60$ y.o.) in urban communities 72.5±5.9 802 (77%) 23.4±3.2	Accelerometer (uniaxial) Right hip Consecutive 7 days Wear time (min): 875.3±92.4 SB (min/d): 801.3±92.4 LPA (min/d): 57.1±22.7 MVPA (min/d): 17.6±15.3	5STS (s): NR TUG (s): NR
Park, 2017 [39]	UK	Older adults (65-99 y.o.), from assisted living facilities, walking unaided 77.5±8.2 85 (68.2%) 28.2±4.9	GT3X+ Right hip A minimum of 3 days Wear time (min): 722.8±68.7 SB (min/d): 511.9±105.7 LPA (min/d): 201.1±72.0 MVPA (min/d): 9.7±9.6	Grip strength (kg): 21.45± 10.85 TUG (s): 13.58±7.40
Rava, 2018 [37]	Estonia	Community-dwelling older adults ($\ge 65$ y.o.), living independently, weight stable over the last six months, not have any cardiac illnesses, neurological illnesses, joint replacements 73.1±5.3 81 (100%) 27.5±4.5	ActiGraph Right hip Consecutive 7 days SB (min/d): 605.5±106.5 LPA (min/d): 261.0±69.7 MVPA (min/d): 56.2±29.6	5STS (s): 9.6±2.0 6MWT (m): 546.0±82.1 TUG (s): 6.0±0.8
Savikangas, 2020 [33]	Finland	Community-dwelling older adults (70-85 y.o.), able to walk 500 m independently, sedentary 74.4±3.8; 293 (58.4%); 27.9±4.8	RM42 Accelerometer (Triaxial) Right iliac crest Consecutive 7 days Wear time (min): 846.0±78.0 SB (min/d): 602.3±82.9 LPA (min/d): 210.3±66.3 MVPA (min/d): 32.5±20.1	6MWT (m): 477.55±82.56 10-meter fast walk (m/s): 1.98±0.38
Silva, 2019 [34]	Portugal	Community-dwelling older adults (65-87 y.o.), from senior universities and daycare centres, non-institutionalized 72.1±5.6; 83 (67.5%); 28.5±4.0	ActiGraph Right iliac crest Consecutive 5 days Wear time (min): 782.5±80.6 SB (min/d): 458.1±78.7 LPA (min/d): 291.2±91.2 MVPA (min/d): 33.5±27.3	6MWT (m): 482.25±98.60 30 s chair stand (n): 15.04 ±5.06 TUG (s): 6.22±2.25
Ward, 2014 [41]	USA	Community-dwelling older adults, without major diseases 68.9±6.7 156 (45.5%) 26.08±4.44	ActiGraph (Triaxial) Non-dominant hip A minimum of 5 days MVPA (min/d): 12.6±13.5	30 s chair stand (n):16.70 ±5.13 6MWT (m): 598.49±107.53 TUG (s): 5.99±1.67 7-meter usual walk (s): 5.43 ±1.98 7-meter fast walk (s): 3.65 ±0.72

*Abbreviations*: *5STS* Five-time sit-to-stand test, *6MWT* six-minute walk test, *BMI* body mass index, *IQR* interquartile, *KE* knee extension, *KF* knee flexion, *LPA* Light-intensity physical activity, *MVPA* moderate-to-vigorous-intensity physical activity, *Nm* newton-meter, *NR* not report, *SB* sedentary behavior, *SPPB* short physical performance battery, *TUG* timed up-and-go test, *y.o.* year-old

### Quality assessment of included studies

Table 3 presents the methodological quality scores of included studies. The JBI score of studies ranged from 62.5% to 100%. Overall, the 13 included studies had an average score of 71%, indicating high quality. Methodological bias mainly was identified in no description of study period and/or location, or no confounding factors being considered, or no detail about the strategies to deal with confounding factors.

**Table 3:** Results of quality assessment of included studies

Study	Item-1	Item-2	Item-3	Item-4	Item-5	Item-6	Item-7	Item-8	Score	Level
Abe, 2012 [45]	Y	N	Y	Y	N	N	Y	Y	62.5	Medium
Aoyagi, 2009 [43]	Y	Y	Y	Y	Y	Y	Y	Y	100.0	High
Davis, 2014 [42]	Y	N	NR	Y	Y	N	Y	Y	62.5	Medium
Duck, 2019 [36]	Y	N	Y	Y	N	N	Y	Y	62.5	Medium
Gerdhem,2008 [44]	Y	Y	Y	Y	N	N	Y	Y	75.0	High
Ofei-Dodoo, 2018 [38]	Y	N	Y	Y	N	N	Y	Y	62.5	Medium
Orwoll, 2019 [35]	Y	Y	Y	Y	N	N	Y	Y	75.0	High
Osuka, 2015 [40]	Y	Y	Y	Y	N	N	N	Y	62.5	Medium
Park, 2017 [39]	Y	N	Y	Y	N	N	Y	Y	62.5	Medium
Rava, 2018 [37]	Y	N	Y	Y	Y	Y	N	Y	62.5	Medium
Savikangas, 2020 [33]	Y	N	Y	Y	Y	Y	N	Y	75.0	High
Silva, 2019 [34]	Y	N	Y	Y	N	N	Y	Y	62.5	Medium
Ward, 2014 [41]	Y	N	Y	Y	Y	Y	N	Y	75.0	High

*Abbreviations*: *Y* yes, *N* no, *NR* not reported

Item 1: Were the criteria for inclusion in the sample clearly defined?

Item 2: Were the study subjects and the setting described in detail?

Item 3: Was the exposure measured in a valid and reliable way?

Item 4: Was objective, standard criteria used for measurement of the condition?

Item 5: Were confounding factors identified?

Item 6: Were strategies to deal with confounding factors stated?

Item 7: Were the outcomes measured in a valid and reliable way?

Item 8: Was appropriate statistical analysis used?

### Association of physical activity’s characteristics and physical fitness

#### Sedentary behavior (Fig. 3)

**Fig. 3:**
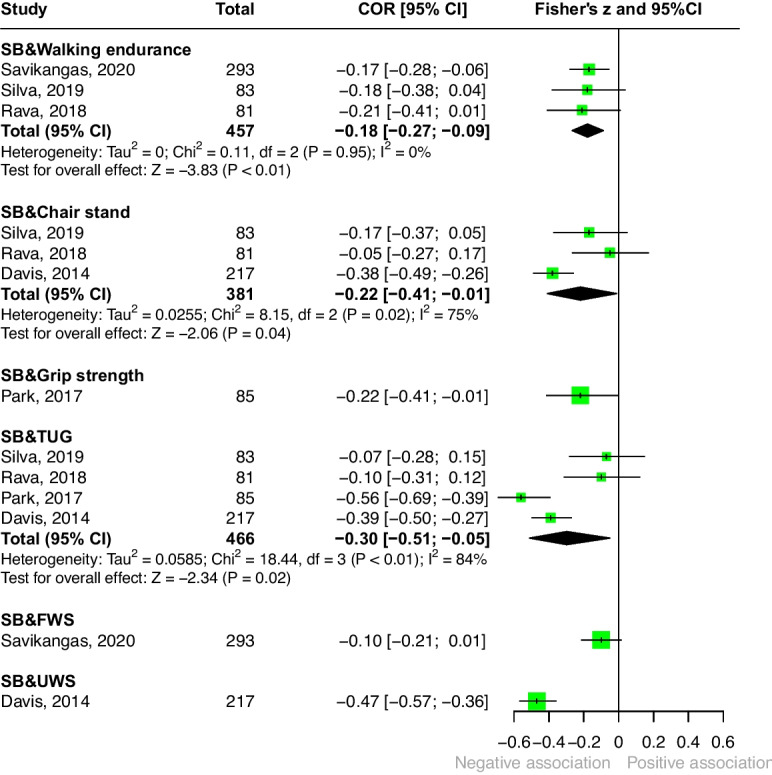
Associations between sedentary behavior and physical fitness. (Abbreviations: FWS = fast walking speed; SB = sedentary behavior; TUG = timed up-and-go test; UWS = usual walking speed)

Five studies reported the associations between sedentary behavior and physical fitness [33, 34, 37, 39, 42]. The pooled correlations indicated weak to moderate negative associations. The moderate correlation was observed for the TUG ($r = -0.30$), followed by leg strength measured by the chair stand test ($r = -0.22$), and walking endurance ($r=-0.18$). Single-study estimates were reported for usual walking speed ($r=-0.47$), grip strength ($r=-0.22$), and fast walking speed ($r=-0.10$). Detailed results are presented in Fig. 3.

#### Light-intensity physical activity (Fig. 4)

**Fig. 4:**
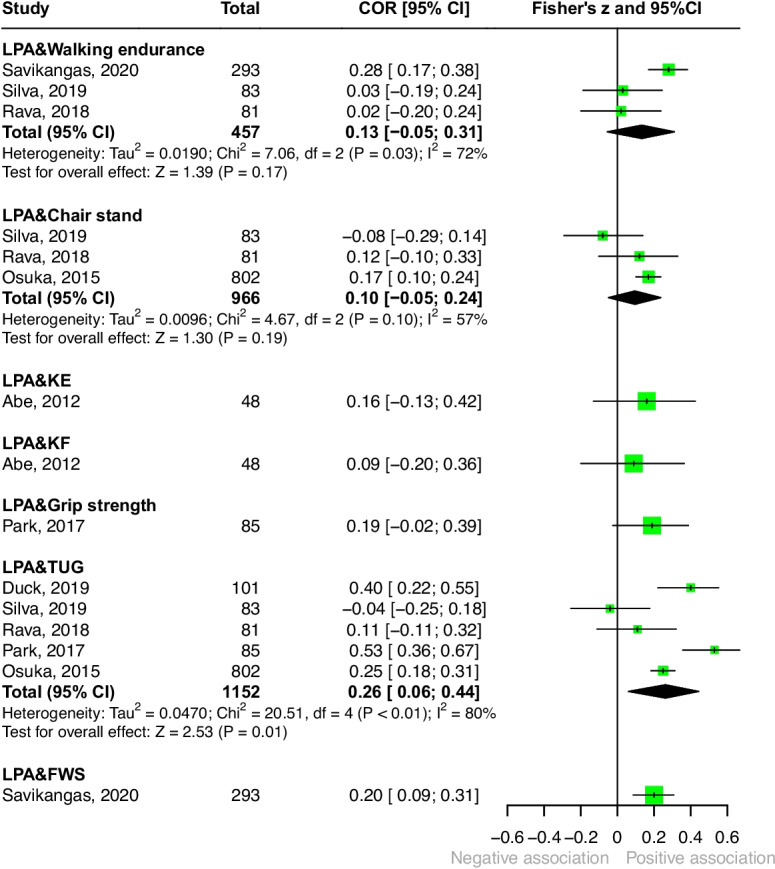
Forest plot of the associations between light-intensity physical activity and physical fitness. (Abbreviations: FWS = fast walking speed; KE = knee extension; KF = knee flexion; LPA = light-intensity physical activity; TUG = timed up-and-go test)

Seven studies reported weak associations between time spent in light-intensity physical activity and physical fitness [33, 34, 36, 37, 39, 40, 45]. A weak correlation was observed for the TUG ($r = 0.26$), followed by chair stand test ($r = 0.10$) and walking endurance ($r = 0.13$). Single-study estimates were reported for fast walking speed ($r = 0.20$), grip strength ($r = 0.19$), and knee extension and flexion strength ($r = 0.16$ and $r = 0.09$, respectively). Detailed results are presented in Fig. 4.

**Fig. 5:**
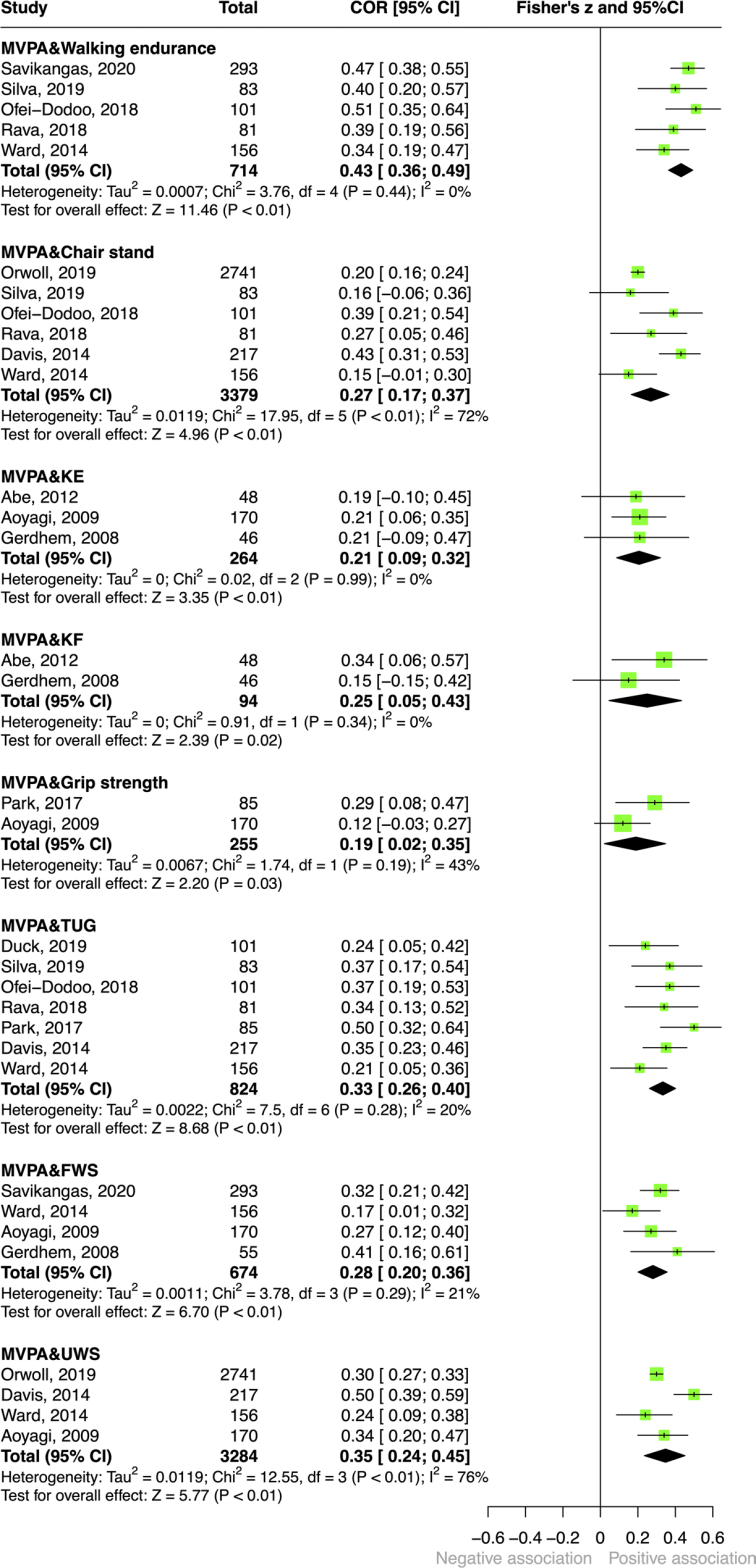
Forest plot of the associations between moderate-to-vigorous-intensity activity and physical fitness. (Abbreviations: FWS = fast walking speed; KE = knee extension; KF = knee flexion; MVPA = moderate-to-vigorous-intensity physical activity; TUG = timed up-and-go test; UWS = usual walking speed)

#### Moderate-to-vigorous-intensity physical activity (Fig. 5)

Thirteen studies examined associations between moderate-to-vigorous physical activity and physical fitness [33–45]. Pooled correlations between moderate-to-vigorous-intensity physical activity and fitness tests ranged from weak to moderate (0.19–0.43). The moderate correlations were observed for the six-minute walk test ($r=0.43$), usual walking speed ($r=0.35$), and TUG ($r=0.33$), while the weak correlations were observed for outcomes in fast walking speed, chair stand test, knee flexion, knee extension, and grip strength ($r=0.19$−0.28). Detailed results are presented in Fig. 5.

### Associations in active vs. inactive older adults

There were four studies with active participants [34, 35, 37, 45] and nine studies with inactive participants [33, 36, 38–44]. In studies with active participants, neither sedentary behavior nor light-intensity physical activity was associated with declines or improvements in physical fitness. While in studies with inactive participants, both sedentary behavior and light-intensity physical activity were related to physical fitness. It showed that sedentary behavior was associated with poorer fitness outcomes in walking endurance ($r=-0.17$) [33], chair stand ($r=-0.38$) [42], grip strength [39], balance ($r=-0.39$ to $-0.56$) [39, 42], and usual walking speed ($r=-0.47$) [42]; and light-intensity physical activity was associated with better fitness outcomes in walking endurance ($r=0.28$) [33], chair stand ($r=0.17$) [40], and TUG ($r=0.25$ to 0.53) [36, 39, 40].

### Publication bias and small study effects

The funnel plots (Supplementary D) for the meta-analysis of the association between light-intensity physical activity and walking endurance showed a slight asymmetry, with a tendency for smaller studies to report more positive effects. The result was supported by Egger’s test, which indicated significant asymmetry (p >0.001). Egger’s test could not be performed for pooled analyses with fewer than two studies. Other pooled analyses showed no small study effects ($p < 0.05$)

## Discussion

This systematic review represents the first comprehensive examination of sensor-based assessments of daily physical activity patterns and their associations with physical fitness in the older population. Our findings indicate that older adults maintaining independent mobility spend an average of 11 hours/day in sedentary behavior, accumulate light-intensity physical activity for approximately 135 minutes/day, and engage in moderate-to-vigorous-intensity physical activity for about 57 minutes/day. The analyses revealed robust inverse associations between sedentary time and multiple physical fitness parameters, including grip strength, chair stand performance, walking endurance, mobility function, and gait speed (both usual and fast-paced). Consistent with existing evidence, but showing more modest relationships than previously assumed, moderate-to-vigorous-intensity physical activity demonstrated positive associations with musculoskeletal fitness (grip strength, knee muscle strength, leg power) and mobility outcomes (walking endurance, mobility function, gait speed), with a moderate correlation coefficient of 0.43 for walking endurance. Notably, light-intensity physical activity showed selective associations with mobility function and gait speed.

A key finding emerging from the cross-sectional evidence is that light-intensity physical activity shows positive associations with physical fitness indicators across multiple domains, including mobility function and gait speed. These measures reflect integrated motor skills requiring strength, balance and endurance [46], while also serving as important clinical indicators of independence [47, 48] and fall risk [49, 50]. Although the relationships were generally low to moderate across all cohorts of older people studied, this review suggests the potential associations between engaging in light-intensity physical activity and these outcomes. In particular, inactive adults exhibit associations with light-intensity physical activity that are comparable to those observed between moderate-to-vigorous-intensity physical activity and physical fitness across all cohorts. Given that light-intensity physical activity constitutes a larger proportion of older adults’ waking time compared to moderate-to-vigorous-intensity physical activity, the maintenance of light everyday activities such as washing the laundry, gardening, or targeted training of these activities (e.g., balance and strength exercise) that can be integrated into everyday life, is recommended. Previous studies have demonstrated the efficacy of this approach. For instance, a study involving 92,541 participants reported a 14% reduction in mortality risk when 30 minutes of daily sitting was replaced with light-intensity physical activity among the least active participants [51]. Additionally, another study indicated that substituting sitting with light-intensity activities (e.g., standing, slow walking) is linked to cardiometabolic benefits comparable to vigorous-intensity exercise, while inducing less exertion in patients with sclerosis [52]. Thus, promoting light-intensity physical activity or functional exercise may serve as an alternative or interim strategy to enhance the well-being of older adults, particularly those with limited capacity for moderate-to-vigorous-intensity activities who still wish to mitigate the adverse effects of sedentary behavior or age-related functional decline and gain health benefits from physical activity or functional exercise. This underlines the importance of implementing programmes that minimize access barriers, including home-based and/or digital training programmes.

This assertion is further supported by findings from studies involving active and inactive participants. The review found that inactive individuals showed particular sensitivity to both sedentary behavior and light-intensity physical activity, reinforcing the"every step counts"principle for those not meeting moderate-to-vigorous-intensity physical activity guidelines. In contrast, active individuals meeting recommended activity levels showed no significant associations between light-intensity physical activity and fitness. Moreover, the associations between moderate-to-vigorous-intensity physical activity and fitness were consistently moderate and stronger than those observed for light activity or sedentary behavior. This distinction highlights that light activity may be closely related to physical fitness in sedentary older adults who have not yet achieved recommended activity levels. Therefore, lower sedentary behavior and more engagement in light-intensity physical activity levels may be associated with better physical fitness, especially in the areas of walking endurance, mobility function, and leg power among less active individuals.

Of particular concern is the alarming sedentary pattern observed among older adults. Although the correlations between sedentary behavior and various mobility measures were weak to moderate, these findings suggests particular association with poorer mobility function which is a robust predictor of falls in this population [53–55]. Mobility impairment, including balance and walking problems ($\beta$=−0.38, $p < 0.01$), have been identified as an important predictor of falls in older adults [55]. While the strength of associations and causal inferences remain limited, the observed trends suggest that interventions aimed at reducing sedentary behaviors and enhancing mobility function may be beneficial. Practical strategies could include encouraging older adults to perform standing chores (e.g., folding laundry) during TV time, incorporate brief stretching sessions or balance exercise every 30-60 minutes of sitting, and use postural reminders (e.g., smartphone alerts).

An unexpected finding was the apparently high moderate-to-vigorous-intensity physical activity levels across studies, though this warrants cautious interpretation. Four studies reported values exceeding WHO’s 150-minute threshold [34, 35, 37, 45], while nine fell below [33, 36, 38–44]. Excluding two outliers [35, 37] with exceptionally active community-dwelling samples reduced the pooled mean to 133 minutes/week. Furthermore, the conflation of moderate and vigorous intensities likely inflates estimates, as vigorous activity constituted $< 5\%$ of total moderate-to-vigorous-intensity physical activity time in most cohorts [33, 36, 37, 45]. Despite these considerations, the results remain promising. This study found positive associations between moderate-to-vigorous-intensity physical activity and physical fitness in older adults, particularly in terms of walking endurance. Compared to light-intensity physical activity and sedentary behavior, moderate-to-vigorous-intensity physical activity exhibits a relatively stronger positive association with walking endurance, an important indicator of cardiopulmonary fitness. A decline in walking endurance predisposes individuals to an increased risk of cardiovascular disease and mortality [56, 57]. These findings align with those of Stamatakis et al. (2022), who reported that even small amounts of vigorous-intensity physical activity, as little as 4 minutes per day, are linked to a 26% to 30% reduction in mortality risk [58]. While light physical activity serves as a prerequisite component for maintaining physical fitness, even brief periods of vigorous physical activity may act as a potent enhancer, being associated with reduced risk of mortality and disease. Based on these findings, regular participation in moderate-to-vigorous-intensity physical activity may be associated with counteracting the detrimental patterns of sedentary behavior, given its closer and more positive relationship with physical fitness. These findings further support the WHO’s recommendation that regular moderate-to-vigorous aerobic exercise is associated with better physical fitness [2]. They underscore the importance of future initiatives aimed at promoting physical fitness among older adults. Health care professionals should provide behavioral interventions to motivate older adults to maintain or increase their levels of moderate-to-vigorous-intensity physical activity, regardless of whether they are engaging in structured exercise programs or daily living activities.

### Limitations and future perspectives

This systematic review, however, has several limitations. First, most included studies were conducted in high-income countries—namely the United States, Japan, and several European nations (e.g., Finland, the United Kingdom)—limiting the geographic and cultural diversity of the evidence base. The underreporting of key sociodemographic variables such as education, income, and ethnicity poses a challenge in assessing potential selection bias and limits the generalizability of findings to broader or underserved older adult populations. Participants enrolled in physical activity studies may also be more health-conscious or mobile than the general population, potentially leading to overestimation of the associations between physical activity and physical fitness. Second, while we focused on sensor-based assessments to capture physical activity—aiming to mitigate recall bias and social desirability from participants [59, 60]—these measures also have limitations, such as their potential for high responsiveness to activity and the inability to monitor certain activities (e.g., aquatic exercise, manual labor, or cycling) [61], which could lead to an inaccurate representation of actual physical activity levels. Despite the overall high quality of the studies, these limitations may compromise the validity of the results. Furthermore, while our analysis employed standardized MET thresholds to classify activity intensities, emerging evidence suggests these criteria may not fully capture perceived exertion in frail or mobility-impaired older adults. For instance, activities classified as light-intensity (1.6–3 METs) by accelerometry—such as folding laundry or slow walking—may require moderate-to-vigorous effort for individuals with severe functional limitations [62]. This misalignment arises because MET values assume a linear relationship between energy expenditure and fitness capacity, which fails to account for age-related declines in cardiorespiratory efficiency [63]. For older adults, the ability to perform essential daily activities—such as walking to a doctor’s appointment or attending a social event—may be of greater relevance than the metabolic intensity of these tasks. Moreover, waist- or hip-worn sensors often misclassify seated activities involving upper-body movements (e.g., playing cards, light crafts) as sedentary due to their limited ability to detect non-ambulatory arm motions. For instance, waist-worn accelerometers may inaccurately classify standing activities as sedentary because they cannot distinguish between sitting and standing postures, leading to potential misclassification of light-intensity activities as sedentary behavior. This limitation underscores the importance of incorporating qualitative contextualization in physical activity research. Lastly, this review focused exclusively on cross-sectional studies, which provide valuable insights into associations between physical activity and physical fitness at a specific point in time, offering a broad overview of trends. However, this design inherently limits our ability to establish causality, as it does not track changes over time. Furthermore, the relationship between physical activity and physical fitness is bidirectional—reduced fitness may limit the ability to be physically active, just as inactivity may be associated with further declines in fitness. Thus, while these studies are useful for identifying potential relationships, the findings should be interpreted with caution. Future research should conduct longitudinal studies to investigate whether sustained changes in sedentary behavior and light-intensity physical activity are associated with cumulative changes on physical fitness, particularly among groups with varying baseline fitness levels. In addition, future studies should incorporate alternative outcome measures—such as total steps, walking time, or task-specific mobility performance—as meaningful indicators of physical activity, rather than relying solely on intensity-based metrics. Moreover, effort should be made to include more diverse samples and systematically report sociodemographic information to better capture population-level variability and reduce selection bias.

## Conclusion

This review highlights that while older adults remain largely sedentary, both moderate-to-vigorous and light-intensity physical activity show potential associations with better physical fitness outcomes. In light of the considerable heterogeneity across participant characteristics, measurement methods, and outcome assessments in existing studies, firm conclusions remain limited. Still, promoting feasible, low-intensity activities may offer practical value in supporting functional health among older adults, particularly those who are unable to engage in higher-intensity exercise. However, recommendations should remain cautious, as the evidence supporting an association between light-intensity physical activity and physical fitness is currently weak.

## Supplementary Information

Supplementary Material 1.

## Data Availability

No datasets were generated or analysed during the current study.

## Abbreviations

WHO: World Health Organization
PRISMA: Preferred Reporting Items for Systematic Reviews and Meta-analyses
MET: Metabolic equivalent task
JBI: Joanna Briggs Institute
CI: Confidence interval
I^2^: I-square
TUG: Timed up-and-go test
