# Supplementary material for: The association between sensor-based assessments of daily physical activity patterns and physical fitness in older adults: a systematic review and meta-analysis
Source: Eur Rev Aging Phys Act. 2025 Sep 30;22:15. doi: 10.1186/s11556-025-00381-y (PMC12487309; doi:10.1186/s11556-025-00381-y)
Supplement: Supplementary file 1 — Supplementary Material 1. [file 11556_2025_381_MOESM1_ESM.docx]

**Supplementary Materials**

Supplementary A: PRISMA Checklist for Cross-sectional Study.

Supplementary B: Literature search strategy.

Supplementary C: Eligibility criteria.

Supplementary D. Funnel plot of meta-analyses

Supplementary A: PRISMA Checklist for Cross-sectional Study

| **Section/topic** | **#** | **Checklist item** | **Information reported** | | **Location** |
| --- | --- | --- | --- | --- | --- |
|  |  |  | **Yes** | **No** |  |
| **ADMINISTRATIVE INFORMATION** | | | | | |
| **Title** | | | | | |
| Identification | 1a | Identify the report as a protocol of a systematic review |  |  | Not applicable |
| Update | 1b | If the protocol is for an update of a previous systematic review, identify as such |  |  | Not applicable |
| **Registration** | 2 | If registered, provide the name of the registry (e.g., PROSPERO) and registration number in the Abstract |  |  | PROSPERO: CRD42023471302 |
| **Authors** | | | | | |
| Contact | 3a | Provide name, institutional affiliation, and e-mail address of all protocol authors; provide physical mailing address of corresponding author |  |  | P1 |
| Contributions | 3b | Describe contributions of protocol authors and identify the guarantor of the review |  |  | P23 |
| **Amendments** | 4 | If the protocol represents an amendment of a previously completed or published protocol, identify as such and list changes; otherwise, state plan for documenting important protocol amendments |  |  | Not applicable |
| **Support** | | | | | |
| Sources | 5a | Indicate sources of financial or other support for the review |  |  | P23 |
| Sponsor | 5b | Provide name for the review funder and/or sponsor |  |  | Not applicable |
| Role of sponsor/funder | 5c | Describe roles of funder(s), sponsor(s), and/or institution(s), if any, in developing the protocol |  |  | Not applicable |
| **INTRODUCTION** | | | | | |
| **Rationale** | 6 | Describe the rationale for the review in the context of what is already known |  |  | P2-3 |
| **Objectives** | 7 | Provide an explicit statement of the question(s) the review will address with reference to participants, interventions, comparators, and outcomes (PICO) |  |  | P3 |
| **METHODS** | | | | | |
| **Eligibility criteria** | 8 | Specify the study characteristics (e.g., PICO, study design, setting, time frame) and report characteristics (e.g., years considered, language, publication status) to be used as criteria for eligibility for the review |  |  | P3-4 |
| **Information sources** | 9 | Describe all intended information sources (e.g., electronic databases, contact with study authors, trial registers, or other grey literature sources) with planned dates of coverage |  |  | P3 |
| **Search strategy** | 10 | Present draft of search strategy to be used for at least one electronic database, including planned limits, such that it could be repeated |  |  | P3 |
| ***STUDY RECORDS*** | | | | | |
| Data management | 11a | Describe the mechanism(s) that will be used to manage records and data throughout the review |  |  | P4 |
| Selection process | 11b | State the process that will be used for selecting studies (e.g., two independent reviewers) through each phase of the review (i.e., screening, eligibility, and inclusion in meta-analysis) |  |  | P3-4 |
| Data collection process | 11c | Describe planned method of extracting data from reports (e.g., piloting forms, done independently, in duplicate), any processes for obtaining and confirming data from investigators |  |  | P3-4 |
| **Data items** | 12 | List and define all variables for which data will be sought (e.g., PICO items, funding sources), any pre-planned data assumptions and simplifications |  |  | P3-4 |
| **Outcomes and prioritization** | 13 | List and define all outcomes for which data will be sought, including prioritization of main and additional outcomes, with rationale |  |  | P3-4 |
| **Risk of bias in individual studies** | 14 | Describe anticipated methods for assessing risk of bias of individual studies, including whether this will be done at the outcome or study level, or both; state how this information will be used in data synthesis |  |  | P5 |
| ***DATA*** | | | | | |
| **Synthesis** | 15a | Describe criteria under which study data will be quantitatively synthesized |  |  | P6 |
|  | 15b | If data are appropriate for quantitative synthesis, describe planned summary measures, methods of handling data, and methods of combining data from studies, including any planned exploration of consistency (e.g., *I* ^2^, Kendall’s tau) |  |  | P6 |
|  | 15c | Describe any proposed additional analyses (e.g., sensitivity or subgroup analyses, meta-regression) |  |  | P6 |
|  | 15d | If quantitative synthesis is not appropriate, describe the type of summary planned |  |  | P6 |
| **Meta-bias(es)** | 16 | Specify any planned assessment of meta-bias(es) (e.g., publication bias across studies, selective reporting within studies) |  |  | P6 |
| **Confidence in cumulative evidence** | 17 | Describe how the strength of the body of evidence will be assessed (e.g., GRADE) |  |  | Not applicable |

Supplementary B: Literature search strategy

| Search Flow |  |  |
| --- | --- | --- |
| Database | PubMed |  |
| Steps | Key words | Results |
| 1 | "Physical activit*" [TIAB] OR "physical activity level*"[TIAB] OR "level of physical activity"[TIAB] OR "Daily physical activit*"[TIAB] OR "leisure physical activit*"[TIAB] | 172,980 |
| 2 | "Aerobic capacity"[TIAB] OR "cardiopulmonary fitness" [TIAB] OR "walk test"[TIAB] OR "balance"[TIAB] OR "strength"[TIAB] OR "physical performance"[TIAB] OR "physical capacity"[TIAB] OR "physical function"[TIAB] | 760,804 |
| 3 | Pedometer [TIAB] OR watch[TIAB] OR acceleromet*[TIAB] OR actigraph[TIAB] OR tactical[TIAB] OR sensewear[TIAB] OR inclinom*[TIAB] OR activpal[TIAB] OR "activity monitor" [TIAB] | 49,526 |
| 4 | 1 AND 2 AND 3 | 2,166 |
| 5 | Limited to human, English/Chinese, mean age≥65 | 801 |
|  |  |  |
| Database | WoS |  |
| Steps | Key words | Results |
| 1 | AB = ("Physical activit*" OR "physical activity level*" OR "level of physical activity" OR "Daily physical activit*" OR "leisure physical activit*") OR TI = ("Physical activit*" OR "physical activity level*" OR "level of physical activity" OR "Daily physical activit*" OR "leisure physical activit*") | 193,560 |
| 2 | AB = ("Aerobic capacity" OR "cardiopulmonary fitness" OR "walk test" OR "balance" OR "strength" OR "physical performance" OR "physical capacity" OR "physical function") OR TI = ("Aerobic capacity" OR "cardiopulmonary fitness" OR "walk test" OR "balance" OR "strength" OR "physical performance" OR "physical capacity" OR "physical function") | 2,047,848 |
| 3 | AB = (Pedometer OR watch OR acceleromet* OR actigraph OR tactical OR sensewear OR inclinom* OR activpal OR "activity monitor") OR TI = (Pedometer OR watch OR acceleromet* OR actigraph OR tactical OR sensewear OR inclinom* OR activpal OR "activity monitor") | 144,688 |
| 4 | 1 AND 2 AND 3 | 2,071 |
| 5 | Limited to article, English | 1,961 |
|  |  |  |
| Database | Embase |  |
|  | Key words | Results |
| 1 | ("Physical activit*" OR "physical activity level*" OR level of physical activity OR "Daily physical activit*" OR "leisure physical activit*"):ti,ab | 226,811 |
| 2 | ("Aerobic capacity" OR "cardiopulmonary fitness" OR "walk test" OR "balance" OR "strength" OR "physical performance" OR "physical capacity" OR "physical function"):ti,ab | 901,208 |
| 3 | (Pedometer OR watch OR acceleromet* OR actigraph OR tactical OR sensewear OR inclinom* OR activpal OR "activity monitor"):ab,ti | 62,925 |
| 4 | 1 AND 2 AND 3 | 3,031 |
| 5 | Limited to English/Chinese, humans, mean age≥65, article/article in press | 786 |
|  |  |  |
|  |  |  |
| Database | CINAHL (By EBSCOhost) |  |
| Steps | Key words | Results |
| 1 | "Physical activit*" OR "physical activity level*" OR level of physical activity OR "Daily physical activit*" OR "leisure physical activit*" | 82,234 |
| 2 | "Aerobic capacity" OR "cardiopulmonary fitness" OR "walk test" OR "balance" OR "strength" OR "physical performance" OR "physical capacity" OR "physical function" | 156,147 |
| 3 | Pedometer OR watch OR acceleromet* OR actigraph OR tactical OR sensewear OR inclinom* OR activpal OR "activity monitor" | 25,712 |
| 4 | 1 AND 2 AND 3 | 208 |
| 5 | Limited to Journal, English, mean age≥65, full-text | 43 |
|  |  |  |
| Database | PsycInfo (by ProQuest) |  |
| Steps | Key words | Results |
| 1 | "Physical activit*" OR "physical activity level*" OR level of physical activity OR "Daily physical activit*" OR "leisure physical activit*" | 52,642 |
| 2 | "Aerobic capacity" OR "cardiopulmonary fitness" OR "walk test" OR "balance" OR "strength" OR "physical performance" OR "physical capacity" OR "physical function" | 120,646 |
| 3 | Pedometer OR watch OR acceleromet* OR actigraph OR tactical OR sensewear OR inclinom* OR activpal OR "activity monitor" | 14,251 |
| 4 | 1 AND 2 AND 3 | 336 |
| 5 | Limited to Journal, English, mean age≥65, journal article | 107 |
|  |  |  |
| Database | Scopus |  |
| Steps | Key words | Results |
| 1 | "Physical activit*" OR "physical activity level*" OR level of physical activity OR "Daily physical activit*" OR "leisure physical activit*" | 405,935 |
| 2 | "Aerobic capacity" OR "cardiopulmonary fitness" OR "walk test" OR "balance" OR "strength" OR "physical performance" OR "physical capacity" OR "physical function" | 3,488,351 |
| 3 | Pedometer OR watch OR acceleromet* OR actigraph OR tactical OR sensewear OR inclinom* OR activpal OR "activity monitor" | 183,019 |
| 4 | 1 AND 2 AND 3 | 3,799 |
| 5 | Limited to English, journal, article, adult | 1,592 |

Supplementary C: Eligibility criteria

| Inclusion | Exclusion |
| --- | --- |
| People of age ≥60 years  People living in community care center  People receiving community services | People hospitalized for any physical or mental health condition, including:   - Acute hospital care - Inpatient rehabilitation - Inpatient mental health care |
| Daily physical activity was collected by accelerometer: including sedentary behavior, light-intensity physical activity, moderate-intensity physical activity, vigorous-intensity physical activity | Subjectively measured physical activity |
| Physical fitness, including walking endurance, balance, and walking speed, muscle strength | No reported measures in physical fitness |
| Correlation analysis between daily physical activity and physical fitness | No reported analyses in correlation between daily physical activity and physical fitness |
| Peer-reviewed journal articles written in English | Thesis, abstracts, conference paper, review, commentary, publication in other language |

Supplementary D. Funnel plot of meta-analyses


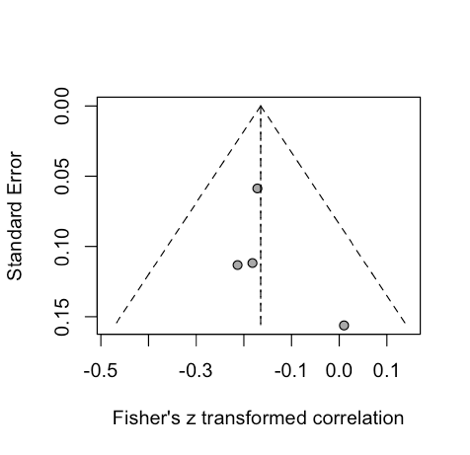


Fig.D1 Correlation between SB and walking endurance


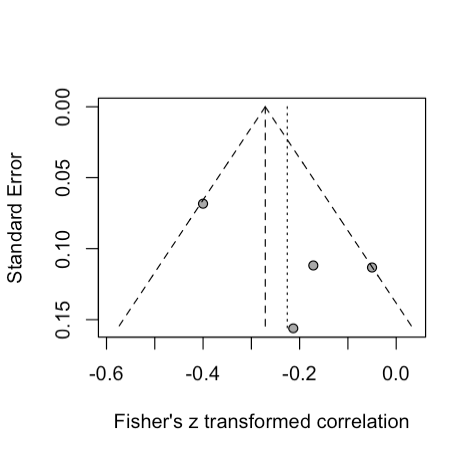


Fig.D2 Correlation between SB and CST

**
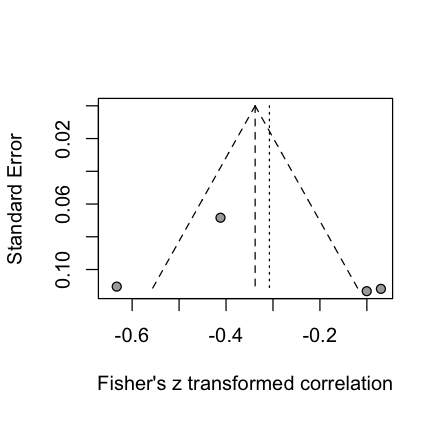
**

Fig.D3 Correlation between SB and TUG


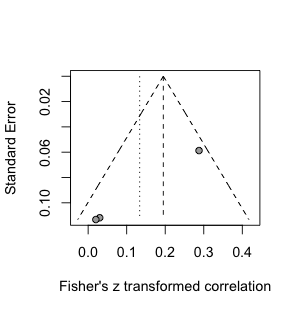


Fig.D4 Correlation between LPA and walking endurance

**
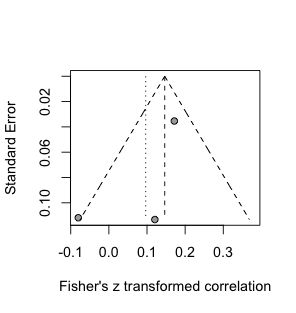
**

Fig.D5 Correlation between LPA and CST


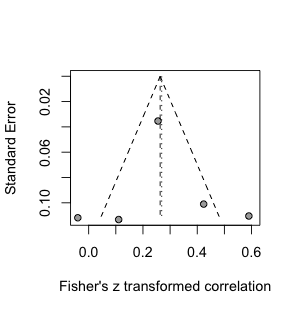


Fig.D6 Correlation between LPA and TUG

**
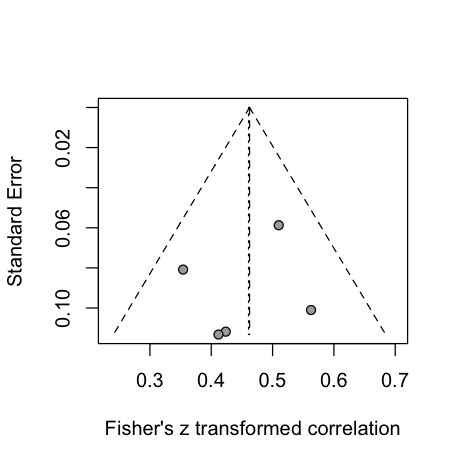
**

Fig.D7 Correlation between MVPA and walking endurance


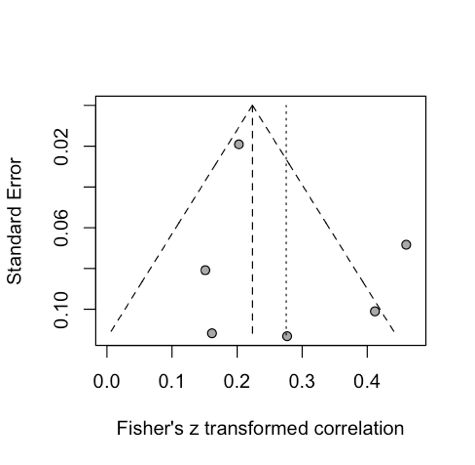


Fig.D8 Correlation between MVPA and CST

**
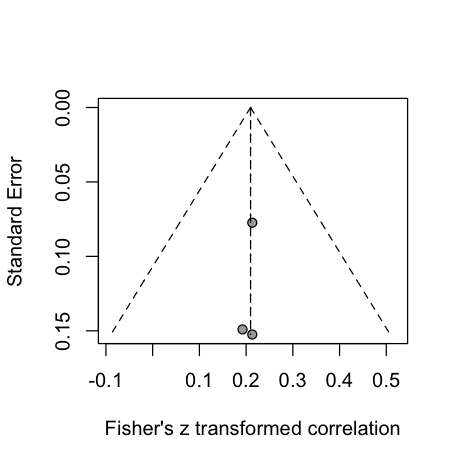
**

Fig.D9 Correlation between MVPA and KE


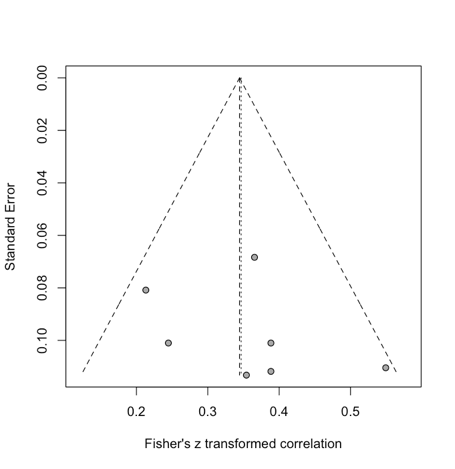


Fig.D10 Correlation between MVPA and TUG


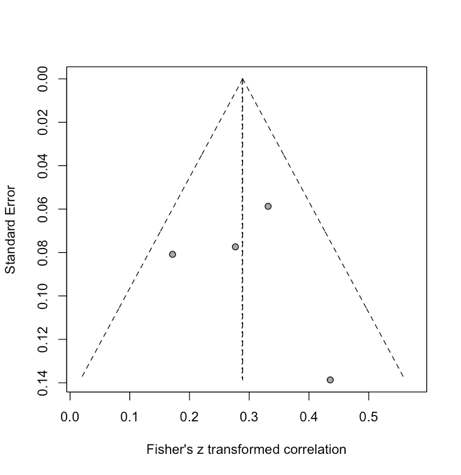


Fig.D11 Correlation between MVPA and FWS

**
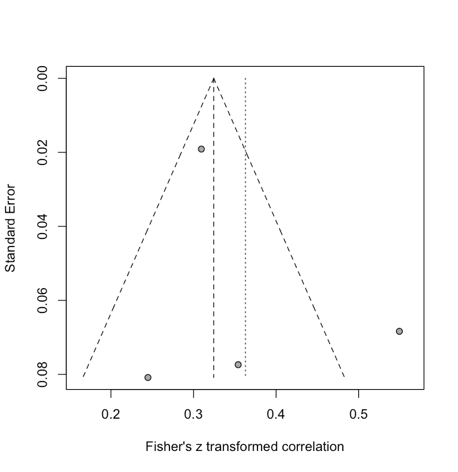
**

Fig.D12 Correlation between MVPA and UWS
